# Proteomic analysis reveals different composition of extracellular vesicles released by two *Trypanosoma cruzi* strains associated with their distinct interaction with host cells

**DOI:** 10.1080/20013078.2018.1463779

**Published:** 2018-04-17

**Authors:** Kleber Silva Ribeiro, Camilla Ioshida Vasconcellos, Rodrigo Pedro Soares, Maria Tays Mendes, Cameron C. Ellis, Marcela Aguilera-Flores, Igor Correia de Almeida, Sergio Schenkman, Leo Kei Iwai, Ana Claudia Torrecilhas

**Affiliations:** a Departamento de Ciências Farmacêuticas, UNIFESP, Diadema São Paulo, Brazil; b Instituto René Rachou/FIOCRUZ – MG, Belo Horizonte, Minas Gerais, Brazil; c Border Biomedical Research Center, Department of Biological Sciences, University of Texas at El Paso (UTEP), El Paso, TX, USA; d Departamento de Microbiologia, Imunologia e Parasitologia, UNIFESP, São Paulo, Brazil; e Laboratório Especial de Toxicologia Aplicada (LETA), Center of Toxins, Immune-Response and Cell Signaling (CeTICS), Instituto Butantan, São Paulo, Brazil

**Keywords:** Extracellular vesicles, *T*. *cruzi* host interaction

## Abstract

*Trypanosoma cruzi*, the aetiologic agent of Chagas disease, releases vesicles containing a wide range of surface molecules known to affect the host immunological responses and the cellular infectivity. Here, we compared the secretome of two distinct strains (Y and YuYu) of *T. cruzi*, which were previously shown to differentially modulate host innate and acquired immune responses. Tissue culture-derived trypomastigotes of both strains secreted extracellular vesicles (EVs), as demonstrated by electron scanning microscopy. EVs were purified by exclusion chromatography or ultracentrifugation and quantitated using nanoparticle tracking analysis. Trypomastigotes from YuYu strain released higher number of EVs than those from Y strain, enriched with virulence factors *trans*-sialidase (TS) and cruzipain. Proteomic analysis confirmed the increased abundance of proteins coded by the TS gene family, mucin-like glycoproteins, and some typical exosomal proteins in the YuYu strain, which also showed considerable differences between purified EVs and vesicle-free fraction as compared to the Y strain. To evaluate whether such differences were related to parasite infectivity, J774 macrophages and LLC-MK2 kidney cells were preincubated with purified EVs from both strains and then infected with Y strain trypomastigotes. EVs released by YuYu strain caused a lower infection but higher intracellular proliferation in J774 macrophages than EVs from Y strain. In contrast, YuYu strain-derived EVs caused higher infection of LLC-MK2 cells than Y strain-derived EVs. In conclusion, quantitative and qualitative differences in EVs and secreted proteins from different *T. cruzi* strains may correlate with infectivity/virulence during the host–parasite interaction.

## Introduction

*Trypanosoma cruzi* cell surface is covered by a wide range of molecules including members of *trans*-sialidase (TS/gp85), mucin-like glycoproteins, mucin-associated proteins (MASP) superfamilies, and proteases [1–3]. Those molecules are involved in attachment and invasion to the host’s cell by infective forms of the parasite [4–6]. Previous reports showed that some of those molecules may act as proinflammatory agents during the innate immune response [7] leading to the production of nitric oxide (NO),IL-6, IL-12, and TNF-α via TLR2 by macrophages [7,8]. Major parasite surface components, such as TS/gp85 glycoproteins, mucins, and surface proteases GP63, were found in extracellular vesicles (EVs) shed by infective trypomastigote forms of the parasite [9,10]. *Trypanosoma cruzi* EVs modulate infectivity, since pre-treatment of BALB/c mice followed by parasite challenge, exacerbated parasite load, heart inflammation, and mortality [11]. Recently, protein and α-galactosyl variations in EVs from different *T. cruzi* strains (Y, Colombiana, CL-14, and YuYu) suggested that intraspecies polymorphisms in those structures could correlate with their ability to induce proinflammatory cytokines [12]. Several reports have shown proteomic variations in different *T. cruzi* stages, including cell derived trypomastigotes and epimastigotes [12–16]. Recently, a proteomic analysis of secreted material released by trypomastigotes obtained from Vero cells infected with CL-Brener and VD strains identified several members of parasite surface molecules [17]. However, a more detailed qualitative and quantitative difference in EVs from *T. cruzi* strains is still unknown.

Here, we performed a quantitative and qualitative analysis of EVs and secreted vesicle-free (VF) fraction of two *T. cruzi* strains (Y and YuYu) classified as TcI and TcII [18], respectively. Those strains displayed significant biological differences regarding infectivity [19], drug resistance [11], and immunomodulation [12]. Purified EVs and soluble fractions from Y and YuYu strains were characterized by liquid chromatography-tandem mass spectrometry (LC-MS/MS)-based proteomic analysis. Further, invasion was assessed after preincubation of EVs in different cells (J774A.1 and LLC-MK2) prior to trypomastigote exposure. Our findings indicate that quantitative and qualitative differences in the EVs may correlate with infectivity for distinct mammalian cells.

## Material and methods

### Ethics statement

The experimental procedures used in this work were approved by the Ethics Committee on Animal Use (CEUA) from the Federal University of São Paulo (http://www.unifesp.br/reitoria/ceua/) protocol #382,321.

### Cell lines and culture

Tissue culture-derived trypomastigotes (TCTs) from YuYu and Y strains of *T. cruzi* were obtained from the culture supernatants of green monkey (Rhesus) kidney LLC-MK2 epithelial cells (ATCC® CCL-7™, Manassas, VA). Cells were maintained in low glucose Dulbecco’s Modified Eagle’s Medium (DMEM) supplemented with 10% foetal bovine serum (FBS; Invitrogen, Carlsbad, CA) at 37°C, 5% CO_2_. Parasites and mammalian cells were regularly tested for *Mycoplasma* [20].

### Preparation of *T. cruzi* EVs and VF fractions

Culture supernatants containing TCTs were centrifuged at 1000 × *g*, 15 min, washed three times in phosphate-buffered saline, pH 7.4 (PBS), and incubated for 2 h in DMEM containing 2% glucose at 37°C, 5% CO_2_ for release of EVs. The parasites (10^9^ trypomastigotes per mL) were removed by centrifugation (1000 × *g*, 10 min, room temperature), and the supernatant (1 mL) was filtered, diluted 1:2 in 200 mM ammonium acetate, pH 6.5, and EVs purified by size-exclusion chromatography (SEC) using a Sepharose CL-4B column (1 × 40 cm, GE Healthcare, Piscataway, NJ), pre-equilibrated with 100 mM ammonium acetate, pH 6.5. After loading the filtered parasite supernatant (1 mL), the column was eluted with the same buffer at a flow rate of 0.2 mL/min. Fractions of 1 mL were collected and then screened by nanoparticle tracking analysis (NTA) and enzyme-linked immunosorbent assay (ELISA) to locate the fractions containing the EVs, as previously described [21].

**Figure 1.**
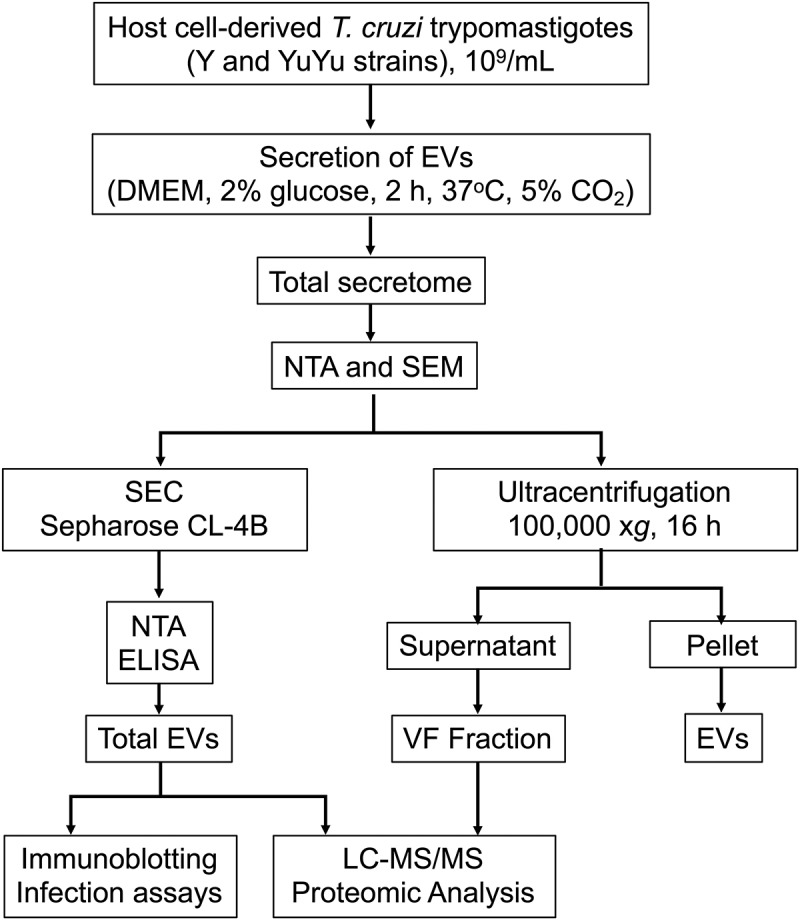
Workflow employed for the production, fractionation, and characterization of *Trypanosoma cruzi* EVs and vesicle-free fraction from Y and YuYu strains. The details of each step are explained in the Materials and Methods section. DMEM, Dulbecco’s Modified Eagle’s Medium; NTA, nanoparticle tracking analysis; SEM, scanning electron microscopy; SEC, size-exclusion chromatography.

Soluble VF fraction was obtained by ultracentrifugation for 16 h at 100,000 × *g*, 4°C, as described [13]. The ensuing supernatant contained soluble, secreted proteins released by the parasite, and the pellet total EVs. For proteomic analysis, pooled preparations of purified total EVs or VF fractions were subjected to (LC-MS/MS), as previously described [10,13], with some modifications (see below). The experimental workflow including the purification and characterization of EVs and VF fraction is depicted in Figure 1.

### Scanning electron microscopy (SEM)

TCT forms from both strains were fixed in a 2.5% glutaraldehyde solution according to established preparation protocols. Briefly, samples were washed in 0.1 M cacodylate solution, fixed with osmium tetroxide, treated with tannic acid, dehydrated with ethanol, and dried in a CPD 030 critical point dryer. The samples were coated with a gold layer using a sputtering method (‘sputtering’, © Leica EM 500 SCD, Germany). Then, samples were observed in a Field Emission FEI Quanta 250 FEG scanning electron microscope [10,21].

### Nanoparticle tracking analysis

EVs from both strains were diluted 100 × in PBS and analysed by using by NTA using a LM10 equipment (Malvern, UK) coupled to a CCD camera and a laser emitting a 60-mW beam at 405-nm wavelength. Data were analysed using Nanoparticle Tracking Analysis (NTA) software (version 2.3 build 0017). The detection threshold was set to 10. ‘Blur’, ‘Min track Length’, and ‘Min Expected Particle Size’ were set to auto. Readings were taken in triplicates during 30 s at 25 frames per second, camera level set to 9, and manual monitoring of temperature (20°C). Differences in concentration and modal size of the EVs were analysed by multiple comparisons and corrected for multiple testing (one-way ANOVA with post hoc Tukey’s test), assuming values of *p* < 0.05 to be significant as described earlier [10].

### Western blotting

Samples containing 30 μg of protein, quantified by BCA technique (Thermo Fisher Scientific), were applied in each lane, resolved in a 12% SDS-PAGE, and transferred to nitrocellulose membranes using standard procedures. Membranes were incubated with anti-*T. cruzi* trypomastigote antibodies [22], monoclonal antibody (mAb) anti-cruzipain (provided by Dr. Ana Paula Lima, Univ. Fed. Rio de Janeiro, Brazil) or mAb 39 anti-TS [23]. Binding was detected after incubation for 1 h at room temperature with the respective peroxidase-conjugated secondary antibody (goat anti-mouse or rabbit IgG) by ECL (Pierce) using an image apparatus.

### Sample preparation for LC-MS/MS analysis

EVs (500 µg of protein) were concentrated in Speedvac for 1–2 h and resuspended in 100 µL of 8 M urea solution containing 100 mM Tris-HCl pH 8 (Kinder and Sherman 2000). The samples were reduced by adding 5 µL of dithiothreitol 1 M to a final concentration of 10 mM followed by 1 h incubation at 56°C. Samples were then alkylated by adding 20 µL iodoacetamide 1 M to a final concentration of 50 mM followed by 1 h incubation at room temperature in the dark. SEC-purified EVs were diluted to 3 volumes by adding 100 mM ammonium acetate and digested with proteomics-grade trypsin (Sigma-Aldrich) at 1:50 ratio of enzyme:sample for 16 h at 37°C. To stop the reaction, 2 µL of glacial acetic acid was added. Digested samples were concentrated in Speedvac (1–2 h), resuspended in 400 µL 0.1% trifluoroacetic acid (TFA), and desalted using Sep-Pak TC18 Light silica-based bonded phase columns (Millipore). The columns were pre-conditioned with 2 mL methanol, 2 mL of 0.1% TFA in 50% acetonitrile (ACN), then 2 mL 0.1% TFA before loading the samples. Then, the columns were washed with 4 mL 0.1% TFA and eluted with 2 mL of 0.1% TFA in 50% ACN.

VF fractions (~10 μg protein) of YuYu and Y strains, obtained as described above, were analysed by high-resolution MS/MS, following digestion of proteins using the filter-aided sample preparation (FASP) method [24], according to the manufacturer’s protocol (Expedeon, San Diego, CA). First, reduction in disulphide bonds of proteins was achieved by treatment with 10 mM dithiothreitol (DTT) for 30 min at room temperature (rt), under constant agitation. Samples were placed on the FASP 30 kDa-cut-off spin filter and centrifuged at 14,000xg for 15 min, at rt. Although the filter cut-off was 30 kDa, we did not observe significant loss of proteins of lower molecular masses (10–30 kDa), using a HeLa whole cell lysate (catalogue # 88,328, Pierce, Thermo Fisher Scientific) and *T. cruzi* trypomastigote lysate as controls (data not shown). Samples were then washed on filter twice with 8 M urea in 50 mM Tris-HCl Buffer (pH 8.0; urea/Tris-HCl buffer, no DTT present), alkylated in the dark for 30 min at rt. Samples were washed again 2 × with the urea/Tris-HCl buffer and 2 × with 50 mM ammonium bicarbonate. A final wash step with 50 mM ammonium bicarbonate was performed prior to transferring the filter to a new tube and incubating with 4 µg proteomics-grade trypsin (Sigma-Aldrich, St. Louis, MO), dissolved in 100 µL 50 mM ammonium bicarbonate, overnight at 37°C, for in-solution digestion, since the proteins were retained but not absorbed to the 30-kDa filter insert. Peptides were eluted from the filter using 200 µL 0.1% formic acid. Samples were dried to ~50-µL volume and subjected to high-resolution LC-MS/MS analysis in a QE Orbitrap (Thermo Fisher Scientific), as described below.

### LC-MS/MS and bioinformatics analysis

Tryptic peptides from EV fractions obtained by SEC were resuspended in 0.1% formic acid and injected into an EASY-nLC II nanoflow liquid chromatography system (Thermo Fisher Scientific) in tandem with an LTQ-Orbitrap Velos mass spectrometer (Thermo Fisher Scientific), at a flow rate of 200 nL/min, in a linear gradient from 5% to 40% mobile phase B (0.1% formic acid in 100% acetonitrile) for 90 min. Samples were separated by capillary reversed-phase C18 fritted-tip analytical column (ID **75** μm × OD 360 μm, 10 cm length), in-house packed with 5-μm Aqua C18 (125Å, Phenomenex, Torrance, CA). Full scans (MS1) were acquired in the Orbitrap analyzer with a resolution of 60,000, at the 300–1800 *m/z* range. The 20 most intense ions were selected for MS/MS (minimum signal 5000) and fragmented by CID (35% power, activation Q 0.250 s, and 10 ms activation time) and detected in the ion trap. For all cluster events, 70 s dynamic exclusion of ions was enabled to minimize repeated fragmentation. Ions clustering for more than once within 25 s were excluded from the selection. Mass spectrum raw files were converted to a peak list format (mgf) by MS Convert software (ProteoWizard), and data processing was performed using Mascot version 2.2 (Matrix Science) against the *T. cruzi* database downloaded from UniProt, with the following parameters: MS1 mass tolerance of 10 ppm, MS2 mass tolerance of 0.5 Da; trypsin as enzyme; carbamidomethylation of cysteine as fixed modification, oxidation of methionine as variable modification. Maximum false peptide and protein discovery rate (FDR) was 1%. Label-free quantitative analysis of Mascot proteomic data was achieved by spectral counting after calculating the exponentially modified protein abundance index (emPAI) for each identified protein [25] (http://www.matrixscience.com/help/quant_empai_help.html). Statistical analysis of the label-free, spectral counting quantitative data was performed by a two-sided Student’s *t* test, with *p* < 0.05 (http://www.matrixscience.com/help/quant_statistics_help.html).

High-resolution MS/MS (HR-MS/MS) analysis of VF fractions of YuYu and Y strains was performed on a Q Exactive Plus Hybrid Quadrupole-Orbitrap MS (Thermo Fisher Scientific), equipped with a Nanospray Flex Ion Source (Thermo Fisher Scientific). Peptides were separated using a Dionex UltiMate 3000 RSLCnano UHPLC system (Thermo Fisher Scientific), with a PicoFrit column (75-μm ID × OD 360 μm, 25-cm length, New Objective, Woburn, MA), in-house packed with a reversed phase Aqua C18 porous silica (5 μm, 125Å, Phenomenex). Before sample injection, the column was equilibrated at a flow rate of 0.5 μL/min with 95% Solvent A (100% H_2_O, 0.1% formic acid) and 5% Solvent B (90% acetonitrile, 0.1% formic acid). Five microlitres of each sample (equivalent to 1 µg protein) was then injected onto the C18 column, and the same equilibration phase was run for 10 min. Elution of the peptides was achieved with a gradient of Solvent B up to 35%, for 85 min, followed by a 5-min increase to 95%, where the plateau was maintained for 9 min. The column was then re-equilibrated to 5% Solvent B for 10 min before injection of the next sample. An automated 2-h run was programmed into Xcalibur software (Thermo Fisher Scientific), and each sample was analysed in technical duplicates with three biological replicates. Between samples, two blank runs with 60-min double seesaw washes using 5–80% ACN gradient to optimally clean the resolving column and limit peptide carryover. During each analysis and all sample runs, the Q Exactive Plus was operated in top-10 data-dependent mode, which comprises 10 data-dependent MS/MS scans for each preceding full scan. The Q Exactive Plus settings were as follows: the normalized collision energy for HCD was 28 eV; a full scan resolution of 70,000, at 400–1600 *m/z* range; a HCD-MS/MS resolution of 17,500 with an isolation width of 3 *m/z*; and dynamic exclusion set at 15 s. Peptides were not excluded based on charge state and 1 microscan for both full and MS/MS scans were acquired. All MS and MS/MS data were acquired in profile mode.

Resulting MS/MS spectra from HR-MS/MS analysis of VF fractions of YuYu and Y strains were searched with Proteome Discoverer (PD) 2.1.1.21 (Thermo Fisher Scientific) and filtered via Percolator with an estimated false-discovery rate (FDR) of 1%. The PD settings were as follows: HCD-MS/MS; cysteine carboxyamidomethylation as fixed modification; methionine oxidation and acetylation on any amino acid as variable modifications; fully tryptic peptides only; up to 2 missed cleavages; parent ion mass tolerance of 10 ppm (monoisotopic); and fragment mass tolerance of 0.6 Da (in Sequest) and 0.02 Da (in PD 2.1.1.21; monoisotopic). Proteins were identified with a minimum of two high-confidence peptides. Tandem MS/MS spectra were searched against a combined protein database of *T. cruzi* (downloaded in FASTA format on 20 January 2017, from UniProtKB; http://www.uniprot.org/; 44,111 entries) and common contaminant sequences (trypsin, human keratins, and protein lab standards, downloaded from http://compbio.ornl.gov/shewanella_chromium_stress/databases/) were included in this combined database. The resulting PD dataset was further processed through Scaffold Q + 4.8.2 (Proteome Software, Portland, OR) to obtain the protein quantification. Files were exported from Scaffold 4.8.2 for further analysis in Scaffold perSPECtives (Proteome Software), where the quantitative analysis (by normalized, weighted spectral counting) and statistical analysis (two-tailed ANOVA, control FDR with standard Benjamin-Hochberg procedure, FDR level *q* = 0.05) were performed. A protein threshold of 99%, peptide threshold of 95%, and a minimum number of 2 peptides were used for identification of proteins. Quantification was performed with normalized weight spectral counts. Statistical analysis was carried out using ANOVA with Benjamin-Hochberg multiple test correction, and significance level of *p* < 0.05. Gene ontology (GO) annotation was carried out using NCBI database (downloaded on 14 February 2017, and 3 October 2017).

### Infection of murine macrophages and epithelial cells

Immortalized murine J774A.1 macrophages (phagocytic) and LLC-MK2 epithelial cells (non-phagocytic) were grown in 24-well dishes on 13 mm circular glass coverslips (Glasscyto) in RPMI and DMEM, respectively. Cells were washed twice with their respective media and were incubated with EVs from Y and YuYu strains (1 and 10 μg protein/well) for 1 h, at 37°C, in RPMI and/or DMEM containing 10% FBS. Cells were infected with TCTs of the Y strain (MOI 1:50), for 2 h at 37°C, and then washed twice with RPMI and/or DMEM without FBS. Non-adherent parasites were removed by two washes with PBS. The cells were then incubated with RPMI and/or DMEM containing 10% FBS for 18 h, at 37°C, or immediately fixed with methanol and stained with 4ʹ,6-diamidino-2-phenylindole, dihydrochloride (DAPI, Molecular Probes, Invitrogen Co., Carlsbad, CA). All experiments were performed in triplicate, and 24 images of each replicate were obtained using a digital video-imaging fluorescent inverted microscope (Nikon), enabling counting of infected and noninfected cells and intracellular amastigotes [26].

## Results

### Fractionation and characterization of *T. cruzi* vesicles

**Figure 2.**
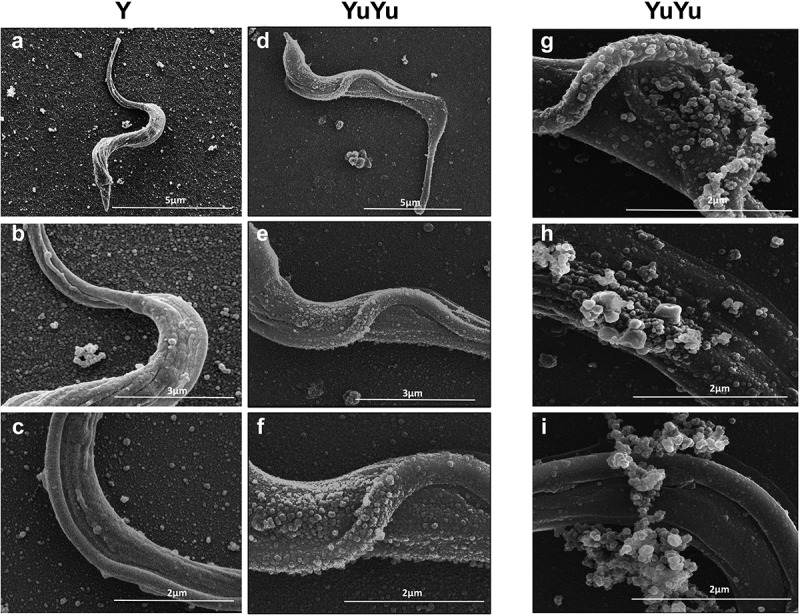
SEM of *T. cruzi* trypomastigotes from Y and YuYu strains showing shedding of EVs. Each panel shows trypomastigotes pre-incubated in DMEM with 2% glucose and attached to glass coverslips containing poly-lysine obtained from the Y (a) and YuYu strains (b), fixed and processed for SEM. The bar sizes are indicated in each image.

Confirming previous results under the same conditions [10], TCTs from each strain were also able to release EVs (Figure 2(a–c)). YuYu TCTs released larger amounts of EVs (Figure 2(d–f)) and putative EVs aggregates (Figure 2(g–i)).

**Figure 3.**
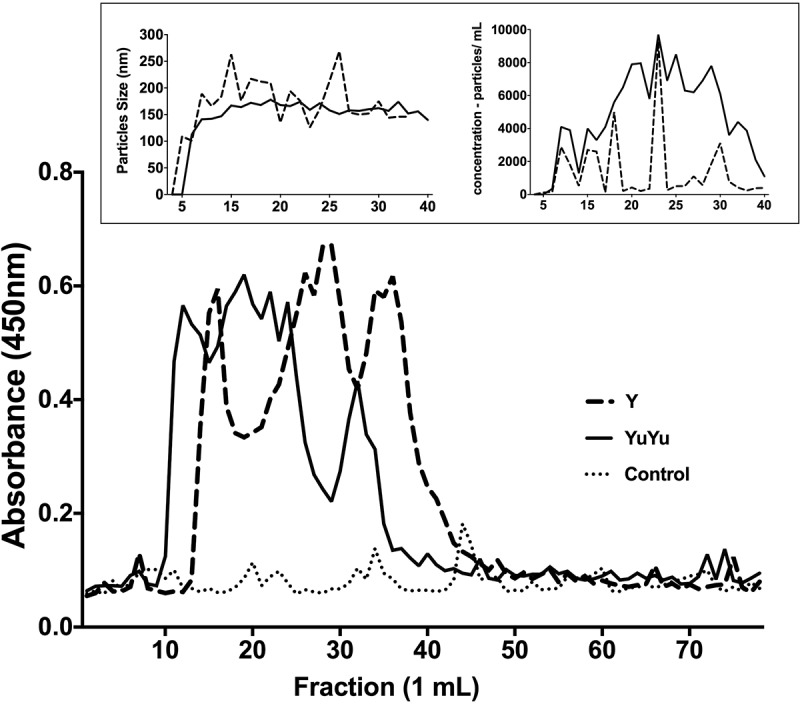
Sepharose 4B elution profile of EVs released by *T. cruzi* trypomastigotes. Total shed vesicles obtained from trypomastigotes (Y, broken lines and YuYu, full line) and control (DMEM and 2% glucose, dotted line) were submitted to gel filtration chromatography using Sepharose CL-4B column and 1 mL fractions were collected. (a) Reactivity of each fraction to antibody 460 was determined by ELISA, and the results are expressed by absorbance at 450 nm. (b) Size (nm) and (c) concentration (particles/mL) of EVs from each fraction as determined by NTA analysis. This figure represents a typical analysis of at least three independent experiments.

After fractionation in Sepharose CL-4B column, EV-containing eluted fractions were probed using total anti-*T. cruzi* antibodies and characterized by NTA. Typical elution patterns of several experiments are represented (Figure 3). EVs from both strains exhibited different size populations in their respective fractions (10–40 mL). Nevertheless, the mean size of the EVs was around 150 nm for both strains (Figure 3, inset, left panel). The mean concentration of particles in the YuYu strain (1.0 × 10^7^–9.7 × 10^9^ particles/mL) was higher than in Y strain (2.7 × 10^6^–8.9 × 10^8^ particles/mL; Figure 3, inset, right panel). Those data confirmed previous SEM results (Figure 2). As expected, no particles were found in the medium alone (negative control; data not shown).

**Figure 4.**
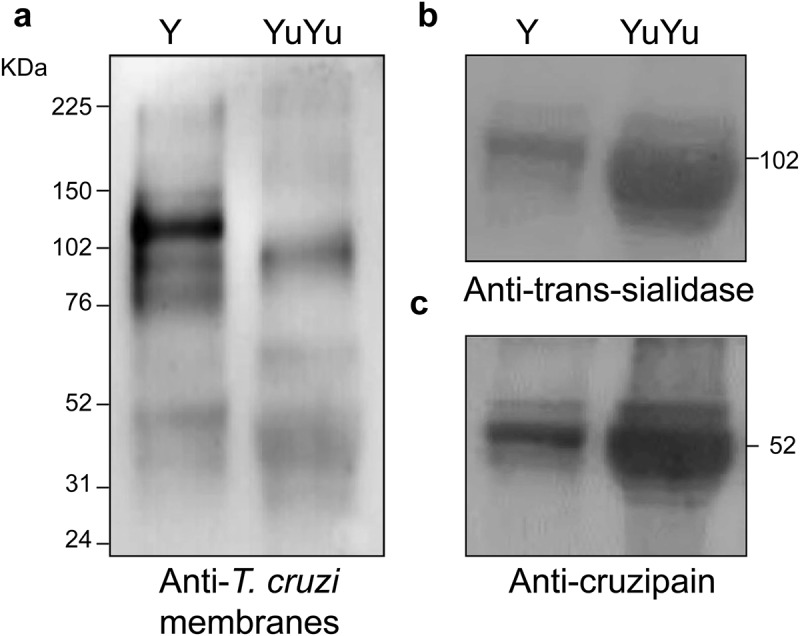
The protein expression profile in EVs is different in Y and YuYu strains of the parasite. Immunoblot of the pooled fractions of EVs released by the Y and YuYu strains probed with antibody 460 (a), ant-TS (b), and anti-cruzipain (c). In the right are indicated the position of mass standards in kDaltons.

EVs from Y strain were highly reactive with anti-α-Gal antibodies from patients with chronic Chagas disease that recognize α-Gal epitopes when compared to those from YuYu strain [10]. Hence, all fractions from each strain were pooled and subjected to immunoblotting to preliminarily assess their composition. EVs isolated from Y strain showed a different labelling with antibody that reacts with total membranes of *T. cruzi* with a predominant reactivity to an 85 kDa band when loading the same amount of protein in the gel (Figure 4(a)). In contrast, YuYu strain showed a higher reactivity for TS (Figure 4(b)) and cruzipain (Figure 4(c)).

### Proteomic analysis

Next, by proteomic analysis, we investigated the compositional differences between YuYu and Y strain EVs, purified by gel-exclusion chromatography on a Sepharose CL-4B column (Figure 1). We also identified the proteins present in the VF fractions obtained by ultracentrifugation at 100,000 × *g*, for 16 h, of conditioned medium from trypomastigotes of Y and YuYu strains, which also might contain proteins weakly associated with EVs (Figure 1). In Supplementary Table 1A, we listed the main proteins identified in the EVs from 5 and 8 biological replicates (biosamples) from EVs of Y and YuYu strains, respectively, obtained by gel-exclusion chromatography, and from 2 and 3 biological replicates (each with two technical replicates) of VF fractions obtained by ultracentrifugation of total secretome of Y and YuYu strains, respectively. The majority of identified proteins corresponded to members of the TS superfamily followed by mucins, MASPs, and surface proteases GP63, which were mainly in the both EV fractions. Proteins from cytoskeletal proteins, such as tubulin, heat shock proteins, and other soluble proteins, were detected in the VF and total EV fractions of both strains and could correspond to vesicle-entrapped and later released proteins, or contaminants of the preparation. At least four major groups (I, II, V, and VIII) [26] of TS proteins were observed, being group V the most abundant in EVs of Y and YuYu strains, in terms of total and average spectral counts (Supplementary Table 1B, underlined numbers).

**Figure 5.**
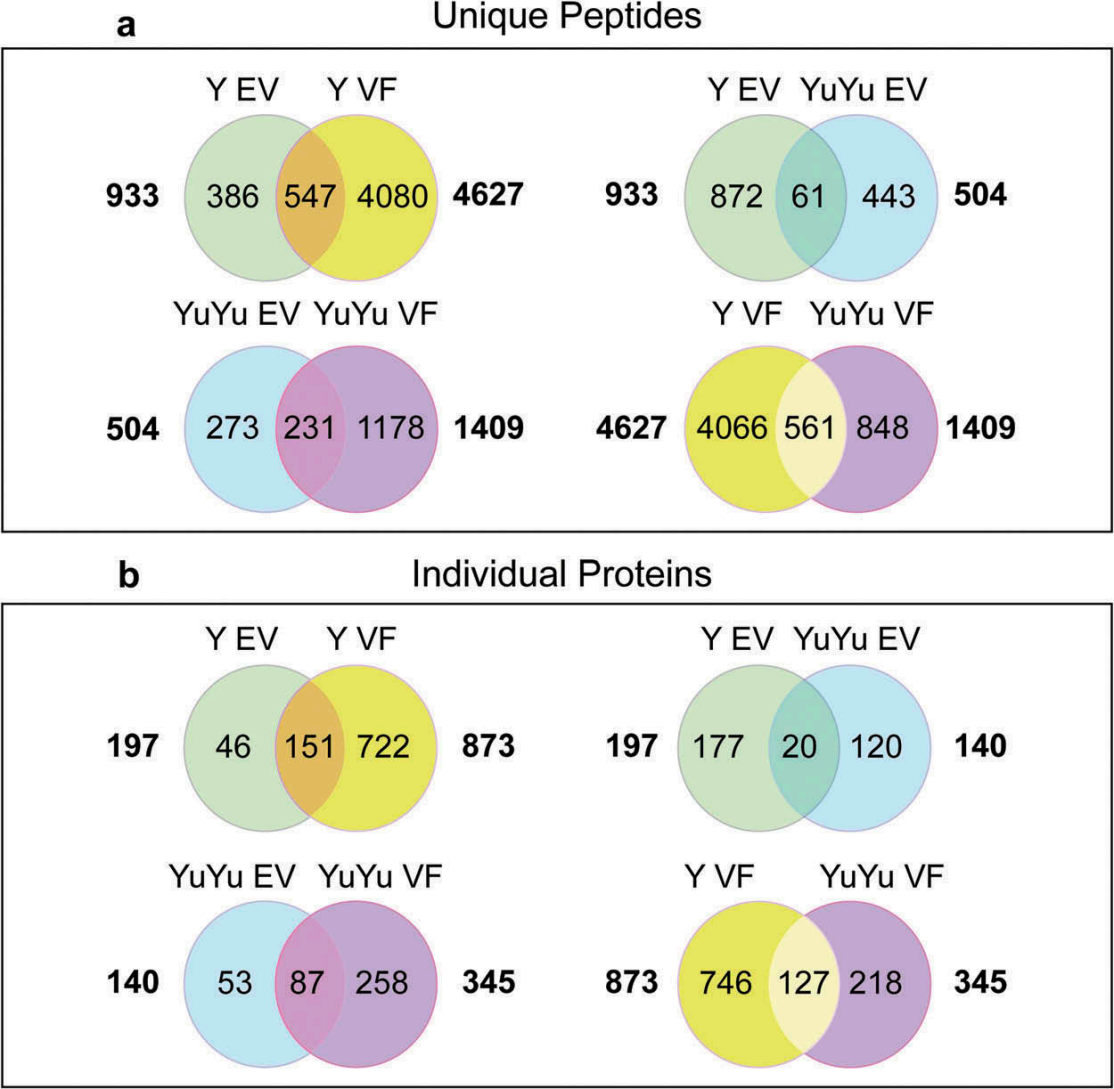
Venn diagrams and cluster analysis of the peptides and proteins detected in EVs from Y and YuYu strains. (a) Analysis of common individual peptides detected in the soluble (VF) and membrane bound fractions (EVs) of the material obtained after gel filtration of the Y (top left) and YuYu strain (bottom left), or between the EVs (top right) and soluble fractions (VF) of the two strains. (b) The same comparison was made using identified proteins.

Venn diagrams in Figure 5(a) show the comparison of similar peptides detected in both the VF fractions and EVs of the two strains. Most of the peptides identified were found in the soluble fraction and were predominant for Y strain. Consequently, a similar number of peptides were detected in the EV fraction of both strains: 933 and 504 in Y and YuYu, respectively. However, only 61 peptides were common between them, suggesting a different composition. When considering the individual proteins identified, the YuYu strain presented at least twice the number of proteins exclusively found in the membrane bound fraction of EVs, when compared to Y strain (Figure 5(b)). Those data confirmed that EVs from YuYu strain are enriched with proteins associated with the membrane. Furthermore, clustering analysis illustrated the differential composition of EVs in both strains and in the supernatant fraction of Y strain. As shown in the summary of the Supplementary Table 1A, more proteins of the TS superfamily and the dispersed gene family 1 (DGF-1) hits were found in the YuYu than in the Y strain. Mucins, mucin-associated proteins, and cysteine peptidases were more detected in the EVs of YuYu and in the VF of Y strain, while the opposite situation was found for the GP63. YuYu also contain more cytosolic and mitochondrial proteins in the EV fraction, suggesting that their vesicles were more stable and do not release their contents during shedding and/or purification processes.

**Figure 6.**
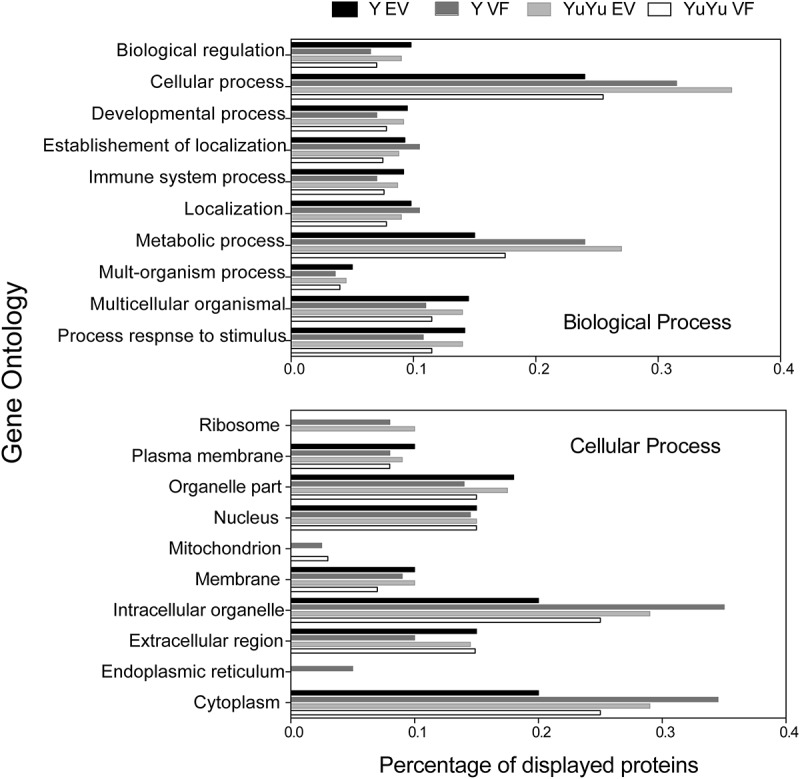
Functional annotation of peptide sequences identified in EVs isolated from Y and YuYu strains of *T. cruzi* using Blast2go software. (a) Biological processes, (b) Cellular components.

The distribution of the proteins based on GO analysis showed larger number of hits in the EVs of YuYu strain for cellular and metabolic processes compared to Y strain (Figure 6). Similar enrichment was observed for proteins located in the cytosol and intracellular organelles, supporting the notion that EVs of YuYu strain encloses more molecules than EVs of the Y strain.

### EVs from different strains cause different infection profiles

**Figure 7.**
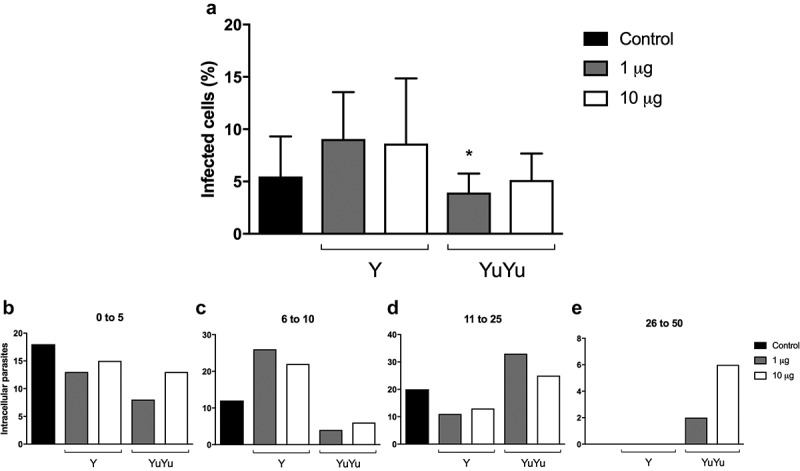
Effect of EVs on macrophages. J774 macrophages (10^5^) were pre-incubated for 1 h with EVs from Y and YuYu strains containing each 1 or 10 µg of protein per mL, equivalent to 10^6^ and 10^7^ or just RPMI medium used as control. Cells were then incubated for 2 h with trypomastigotes (10 parasites/cell), washed and the incubation proceeded for more 24 h. The cells were fixed and stained with DAPI and the number of intracellular parasites determined in cells containing from 0 to 5 parasites/cells (a), 6 to 10 parasites/cells (b), 11 to 25 parasites/cells (c), and 26 to 50 parasites/cells (d). The values are means of duplicate experiments.

**Figure 8.**
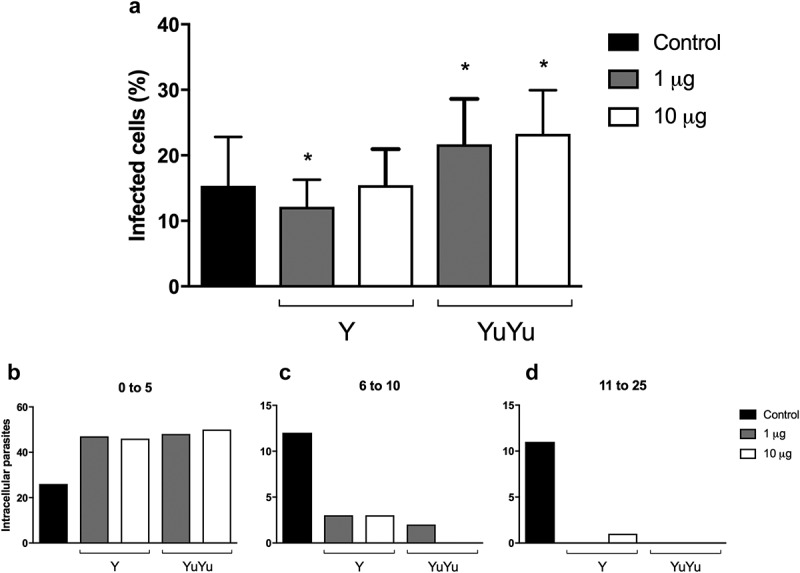
Effect of EVs on macrophages. LLC-MK_2_ (10^5^) were pre-incubated 1 h with EVs from Y and YuYu strains containing each 1 or 10 µg of protein per mL, equivalent to 10^6^ and 10^7^, or just DMEM medium used as control. Cells were then incubated for 2 h with trypomastigotes (10 parasites/cell), washed and the incubation proceeded for more 24 h. The cells were fixed and stained with DAPI and the number of intracellular parasites determined in cells containing from 0 to 5 parasites/cells (a), 6 to 10 parasites/cells (b), and 11 to 25 parasites/cells (c). The values are means of duplicate experiments.

Since main differences in EVs derived from YuYu and Y strains were in proteins known to act during the processes of cell adhesion and infection [5,22,27,28], their role during invasion was evaluated in phagocytic J774A.1 macrophages and non-phagocytic LLC-MK2 cells. Cells were pre-incubated for 1 h with EVs from both strains and then exposed to TCT of the Y strain for 2 h. EVs from Y strain increased the number of J774A.1 macrophages infected with *T. cruzi* (Figure 7(a)). In general, this affect was not dependent on protein concentration (Figure 7(a–e); Figure 8(a–d)). Interestingly, pre-treated cells with YuYu EVs showed larger numbers of intracellular parasites (Figure 7(d-e)). Different from macrophages, in non-phagocytic cells, an increased internalization of trypomastigotes in the presence of EVs from YuYu strain was detected (Figure 8(a)). However, intracellular proliferation of amastigotes was lower than that of phagocytic cells (Figure 8(b–d)).

## Discussion

EVs from *T. cruzi* strains (Y and YuYu), previously known to have different biological and immunomodulation properties [10,29], were evaluated. Also, their soluble proteins (VF) were assessed. Similar to whole parasite, YuYu EVs were enriched with TS family of glycoproteins, mucins, and mucin-associated proteins when compared to those from Y strain. Those molecules are well-established *T. cruzi* virulence factors [1,5,7,8,30–34]. Both strains were able to release EVs suggesting that similar mechanisms of their biogenesis may occur among different *T. cruzi* groups [18,35]. An interesting feature observed for YuYu strain is its ability to release a higher amount of EVs than Y strain as confirmed by SEM and NTA. This feature may be also strain specific since YuYu and Y strains belong to TcI and TcII groups, respectively [18]. Although both EVs showed similar sizes, they exhibited intraspecies polymorphisms in their contents confirming preliminary qualitative analysis [10]. Therefore, the amount and, perhaps its contents, of EVs might be one of the factors involved in infectivity/immunopathology, since those structures contain several pro-inflammatory molecules (see review in [18,29,36]). In order to ascertain possible qualitative features of EVs, a preliminary analysis by immunoblotting was performed.

Consistent with previous observations, a distinct protein profile was observed between the two strains after probing with total anti-*T. cruzi* antibody. EVs from` Y strain appear to be enriched in proteins ranging from 80 kDa to 150 kDa. On the other hand, a major 100-kDa protein was present in YuYu EVs. However, after probing with anti-TS and anti-cruzipain, a higher expression of those proteins was detected for YuYu strain. Those data confirmed that this strain can release more vesicles, and those are enriched with those molecules.

Proteomic analysis of VF and EVs also confirmed that significant qualitative differences exist between strains. Interestingly, some components are more retained in the membrane-associated fractions of YuYu EVs (which also contained several cytosolic proteins), compared to Y. The proteomic analysis further confirmed the enrichment for members of the TS gene family. Several members of this gene family were mainly found in the membrane bound fractions of YuYu-EVs, while most of them were found in the soluble fractions of Y-EVs. Those data are in agreement with previous secretome analysis [37], showing the presence of soluble and membrane bound TS from *T. cruzi* trypomastigotes [13,38].

Most of the detected members of the TS superfamily corresponded to the Group II and V (see Supplementary Table I), which are encoded by the most abundant genes in the genome. These proteins are found in the surface of trypomastigotes [39,40] and contain a glycosylphosphatidylinositol inositol (GPI) anchor [41]. Group V displays only one typical sialidase domain and a modified segment in lectin domain involved in the interactions with host cell through the FLY sequence [5,32]. This group predominated in the YuYu EV fraction. In contrast, we detected the presence of the group II more abundantly in the Y-EVs, which correspond to the 85 kDa family of glycoproteins involved in the parasite interaction with host cells. Those proteins harbour the typical FLY sequence and two sialidase domains [4,5,23,42] and monoclonal antibodies against one set of these proteins, called Tc85, partially inhibits the host’s cell invasion by the parasite [43].

Our analysis also detected more hits for Group I in the YuYu EVs, which correspond to the proteins containing TS activity, in agreement with our Western blot analysis, which used an antibody to recognize the carboxy-terminal domain of the TS repeats, also denominated shed acute phase antigen (SAPA) [23]. The TS enzymes are known to be released by trypomastigotes and influence the host infectivity [12,44,45] and to stimulate the production of IL-6 in intestinal microvascular endothelial cells and peripheral blood mononuclear cells [46]. It is important that some of these proteins were also released in the soluble form as shown before [47]. Interestingly, the SAPA repeats were not largely detected in our analysis since they do not contain lysine residues to generate short peptides to be detected by our MS/MS analysis. We only observed some hits for Group VIII, a less characterized set of abundant genes and no members of Group III, IV, VI, and VII. Group VI comprehends proteins with 160 kDa also involved in the interaction of trypomastigotes with cells [48]. There it is possible that they were expressed in relatively small amounts in trypomastigotes or were not released with the EVs.

We have also identified several peptide sequences corresponding to the mucin-associated surface proteins (MASP), also detected in total proteome of *T. cruzi* trypomastigotes [49]. Most of the MASP hits were found in the EVs from both strains, although different clusters were enriched in each one of them. MASP is a member of a multigenic family, with variable amino acid sequences containing a common N-terminal and a GPI-anchoring sequence [50]. Therefore, these proteins appear to be a principal component of the EVs membranes, differently of the TS family that seems to be more easily released in the soluble form. As the biological function of MASP still remains to be determined, the significance of our findings would require further investigation. In contrast, we did not detect abundant hits for the mucin-like glycoproteins, known to be abundant in the surface of trypomastigotes [1,27]. Previous studies with highly purified tGPI-mucins suggested that these molecules are difficult to analyse by conventional proteomic analysis due to their amino acid composition, sequence diversity, and extensive post-translational modifications [51]. Although the proteomic analysis from all four *T. cruzi* stages was not able to identify those proteins [49], a significant number of hits of TcMUCII mucins were obtained through proteomic analysis of trypomastigotes by 2D LC-MS/MS [49]. A recently discovered family of proteins rich in serine, alanine, and proline (SAP), with a sequence similar to MASP and shown to be expressed and secreted to external medium by metacyclic-trypomastigotes forms, and also to play a role in host cell invasion and Ca^2+^ signalling [52,53], was minorly detected in our fractions.

Similar to *Leishmania* EVs [54], EVs from *T. cruzi* strains also possess gp63, a surface glycoprotein with protease activity. Previous studies already showed that the gp63 from *T. cruzi* has a key role in parasite infection [3] and complement inhibition [55]. We identified 7 clusters of this protein, and most of the hits were found in the EVs from Y strain. Interestingly, for cruzipain, which is a major cysteine proteinase released by trypomastigotes [56], a few hits revealed this protein in our proteomic analysis. It has been reported that cruzipain activity remains in the supernatant of parasite culture medium after high-speed centrifugation at 100,000 × *g* [57]. Secretome analyses of other trypanosomatids [13,14,38] such as *Trypanosoma brucei, Leishmania donovani,* and *Leishmania major* have shown that a large proportion, if not all of the secreted proteins are released as membrane-bound vesicles [58–60], similar to mammalian exosomes. Interestingly, in *L. donovani,* the composition and number of secreted vesicles seems to be regulated by conditions mimicking infection of host cells [60]. Among the protozoan parasites, *Plasmodium* [61] was shown to secrete EVs into the mouse plasma, which contributes to inflammation by activating macrophages [62]. In our study, not only secreted but also membrane bound proteins exhibited intraspecies polymorphisms that could be responsible for the differential abilities of those strains to cause various outcomes in the host during infection. In this sense, the ability of EVs during invasion/proliferation mechanisms was assessed during functional interaction with phagocytic and non-phagocytic cells.

EVs of both strains showed marked differences during the interaction of parasites with host cells. They differentially modulated invasion by trypomastigotes of the Y strain in two different cell lines. J774A.1 macrophages pre-treated with EVs from Y strain showed increased trypomastigote invasion, whereas those from YuYu displayed increased intracellular proliferation. The same situation was not observed in LLC-MK2 cells suggesting that this mechanism may be dependent on cellular type. Consistent with those observations, different mechanisms of cell invasion by *T. cruzi* and the variable responses of macrophages compared to non-phagocytic cells were already reported [63]. Macrophages use phagocytic process to internalize *T. cruzi* and [64] this could be affected EVs. For example, we observed that Toll-like receptors have been shown to modulate invasion by the EVs, which contain GPI-anchors (unpublished results). The growth of the intracellular parasites could be also affected by the different escape mechanisms from the parasitophorous vacuole [65,66], or the specific induction of inflammasomes in the macrophage lines [67]. One hypothesis to explain those polymorphisms is the lower expression of α-galactosyl residues in the YuYu EVs [10] since pre-treatment with tGPI-mucins of Colombiana strain (TcI) resulted in lower ability of infection compared to Y strain (TcII) [26]. Those results could indicate that terminal α-Gal residues are involved in the prominent enhancement of host-cell infection by TCTs mediated by EVs. It is also possible that these differential host cell–parasite interactions induced by trypomastigote-derived vesicles could be determined by distinct EV subpopulations (i.e. exosomes and ectosomes), containing very diverse composition of virulence factors and other host-cell modulators, as previously described for metacyclic trypomastigotes [13]. Future expression and functional proteomics of different EV subpopulations could shed some light on these disparate modulatory activities of trypomastigotes vesicles on various host cells types.

In conclusion, this study assessed proteomic analysis of EVs in different *T. cruzi* strains. Important qualitative differences were found not only in the EVs, but also in their secretome, may be determinant during the host parasite interaction. More importantly, the presence of virulence factors involved in pathogenesis and immunopathology suggests their ability to modulate different host inflammatory immune responses. Finally, those EVs were able to differentially affect invasion/proliferation inside phagocytic and non-phagocytic cells.

## Supplementary Material

Suppl._Tables.zip
